# Thymome médiastinal: un diagnostic à ne pas méconnaitre

**DOI:** 10.11604/pamj.2015.20.409.6119

**Published:** 2015-04-27

**Authors:** Madiha Mahfoudhi, Khamassi Khaled

**Affiliations:** 1 Service de Médecine interne A, Hôpital Charles Nicolle, Tunis, Tunisie; 2 Service d'ORL, Hôpital Charles Nicolle, Tunis, Tunisie

**Keywords:** Thymome, médiastin, dysphonie, chirurgie, thymoma, mediastinum, dysphonia, surgery

## Image en medicine

Les thymomes sont des tumeurs épithéliales rares chez l'adulte. Ils se manifestent le plus souvent par une douleur thoracique, une dyspnée ou un syndrome cave supérieur associés ou non à une myasthénie. La rareté et la diversité de la présentation clinique rend le diagnostic positif des thymomes difficile. Patient âgé de 40 ans, tabagique, qui a consulté pour une dysphonie chronique évoluant depuis 3 mois, qui s'est exacerbée depuis 15 jours sans dyspnée ni dysphagie. L'examen cervical trouvait une loge thyroïdienne et des aires ganglionnaires libres. La nasofibroscopie a révélé une corde vocale gauche immobile en position paramédiane. En laryngoscopie directe, l'endolarynx était d'aspect normal. La Radiographie du thorax était sans anomalies. Le bilan biologique était normal. La TDM thoracique a objectivé un processus tissulaire médiastinal supérieur, de rehaussement intense et hétérogène, mesurant 9 x 5 cm, arrivant en bas jusqu'au toit de la crosse de l'aorte. Le patient a été opéré en chirurgie thoracique par voie de sternotomie et a eu une exérèse totale de la masse. Celle-ci présentait des adhérences avec le nerf récurrent gauche sans l'envahir. Les suites post-opératoires étaient simples. L'examen anatomopathologique a conclut à un thymome encapsulé sans signes histologiques de malignité. L'évolution était marquée par une amélioration de la dysphonie.

**Figure 1:**
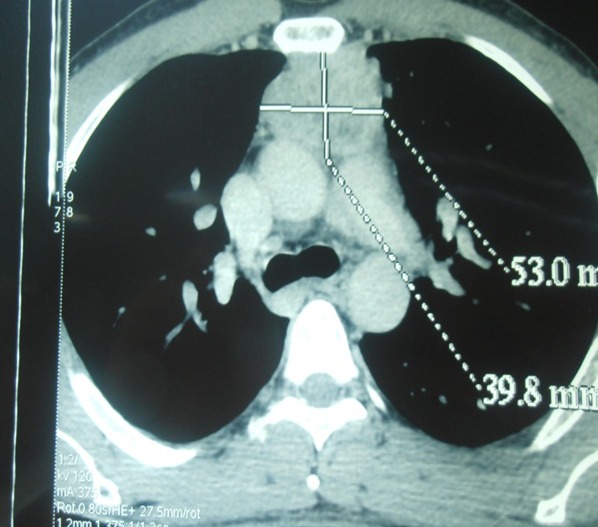
TDM thoracique (coupe axiale): processus tissulaire médiastinal supérieur, de rehaussement intense et hétérogène, mesurant 9 x 5 cm, arrivant en bas jusqu'au toit de la crosse de l'aorte

